# N-coordination-driven anchoring of M_2_ dimers (M = Cu, Ag) on Au_6_ cluster: structural evolution and enhanced photoluminescence

**DOI:** 10.1039/d6sc04505g

**Published:** 2026-08-05

**Authors:** Yukatsu Shichibu, Kazuki Yamada, Hiroshi Itakura, Hiroto Morita, Lan Xu, Katsuaki Konishi

**Affiliations:** a Graduate School of Environmental Science, Hokkaido University North 10 West 5 Sapporo 060-0810 Japan shichibu@ees.hokudai.ac.jp konishi@ees.hokudai.ac.jp; b Faculty of Environmental Earth Science, Hokkaido University North 10 West 5 Sapporo 060-0810 Japan

## Abstract

Precise alloying of gold clusters is a cornerstone for tailoring their structures and properties; however, atomic-level control over heterometal placement remains elusive due to the inherently stochastic nature of conventional synthetic routes. Herein, we report a coordination-driven alloying strategy that enables site-specific grafting of M_2_ dimers (M = Cu, Ag) onto a Au_6_ core stabilized by pyridyl-bridged diphosphines. Single-crystal X-ray analysis reveals that pyridyl-N → M coordination governs the formation of these Au_6_M_2_ clusters, with Au_6_Cu_2_ adopting a markedly more compact framework. While heterometal incorporation slightly perturbs the absorption bands associated with frontier orbitals, it remarkably enhances near-infrared photoluminescence in solution, affording up to a 150-fold increase in quantum yield for Au_6_Cu_2_. Theoretical analyses identify the heterometals as electron-deficient, weakly interacting species, indicating a ligand-governed surface anchoring mode rather than core-level substitution. This work establishes coordination-driven postsynthetic alloying as a powerful and deterministic strategy for atomically precise engineering of gold-based alloy clusters *via* controlled heterometal anchoring.

## Abstract 2

A pyridyl-diphosphine scaffold directs the regiospecific anchoring of M_2_ dimers onto a Au_6_ cluster. This coordination-driven alloying triggers a structural rearrangement and boosts NIR emission by up to 150-fold.

## Graphical abstract

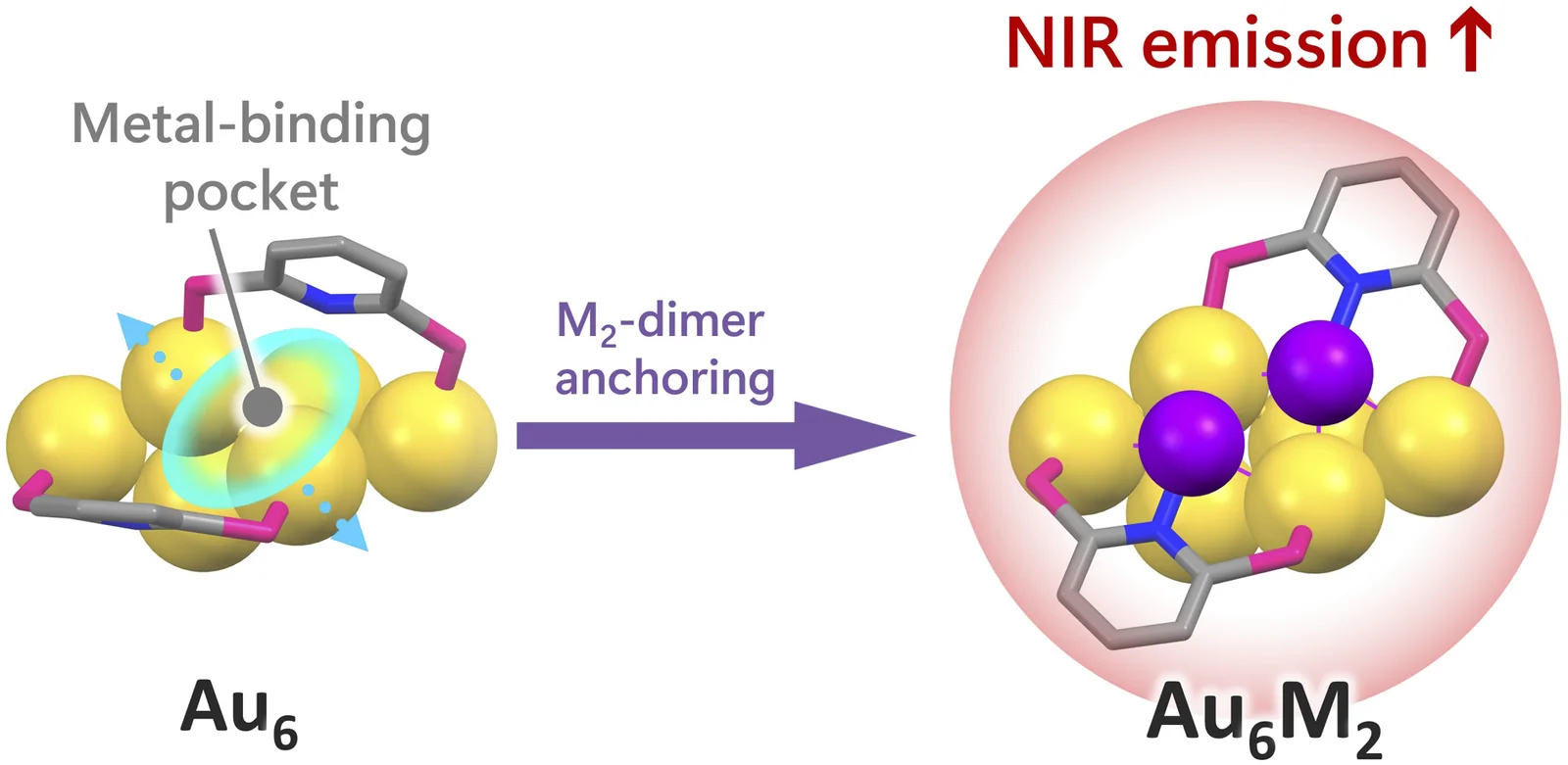

## 1 Introduction

Gold-based alloy clusters, typically stabilized by organic ligands,^1–3^ have emerged as a promising platform for exploring optical^4–7^ and catalytic^8–10^ properties. To control their composition and properties, “substitutional incorporation” has been predominantly employed as a traditional and widely adopted alloying strategy. This involves replacing gold atoms within a predefined framework, targeting specific sites ranging from central doping (*e.g.*, icosahedral M@Au_12_ (ref. 5)) to peripheral substitution (*e.g.*, biicosahedral Au_25−*m*_Ag_*m*_ (*m* = 1–13)^4^). In contrast, “surface anchoring” *via* ligand-mediated heterometal grafting remains less common, with only a few reports demonstrating the use of N-donor monodentate ligands like PPh_2_Py and SPy to incorporate heterometal adatoms onto gold cores such as Au_13_Cu_*m*_ (*m* = 2, 4, 8)^11,12^ and Au_*n*_Ag_2_ (*n* = 6, 10).^13^ Conventionally, gold-based alloy clusters are synthesized *via* co-reduction of gold and heterometal precursors, in which metal-core nucleation and heterometal incorporation occur simultaneously. However, the stochastic nature of this one-pot synthesis limits control over reaction parameters and restricts structural diversity, despite discernible empirical trends. Addressing these limitations requires a rational, bottom-up alloying strategy that decouples heterometal incorporation from metal-core nucleation and exploits ligand–heterometal coordination to direct site-specific alloy formation.

Diphosphine ligands are powerful structural modulators of ultrasmall gold clusters,^14,15^ enabling the tuning of electronic and photophysical properties through ligand-bridge design. For instance, a C3-bridged diphosphine (Ph_2_P-(CH_2_)_3_-PPh_2_) allowed precise control over the nuclearity and optical absorption of prolate-shaped gold clusters.^16–18^ Furthermore, modifications of the alkyl bridge, such as adjusting chain length or introducing aromatic substituents, regulated luminescence,^19–22^ chiroptical activity,^22,23^ and ligand–core interactions.^24^ Recently, a diphosphine bearing a 2,6-dimethyl-substituted pyridyl bridge was shown to facilitate postsynthetic Ag incorporation into a Au_8_ cluster, affording Au_9_Ag_4_.^25^ However, the pyridyl-N sites in those clusters remain uncoordinated due to their spatial separation from the metal core. This suggests that short-linker N-donor diphosphines could position their donor sites in close proximity to gold-core surfaces, thereby promoting heterometal anchoring *via* ligand–heterometal coordination.

**Fig. 1:**
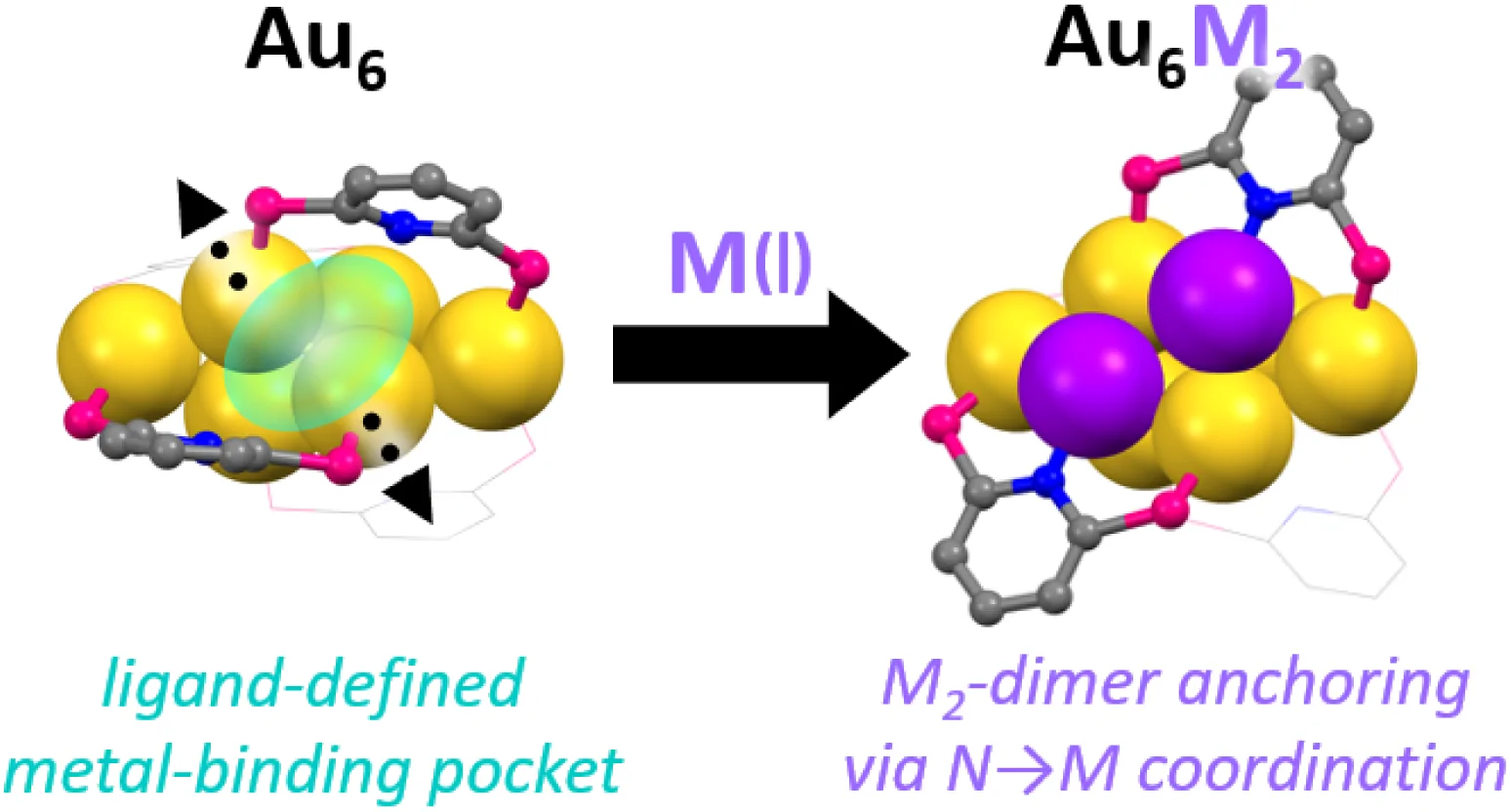
Schematic illustration of the coordination-driven anchoring of M_2_ dimer onto preformed Au_6_ cluster.

Herein, we report a coordination-driven alloying strategy that achieves the site-specific anchoring of M_2_ dimers (M = Cu, Ag) onto a Au_6_ cluster using a pyridyl-bridged diphosphine, 2,6-bis(diphenylphosphino)pyridine (2,6-PyDP) (Fig. 1). This ligand-directed postsynthetic approach effectively decouples heterometal incorporation from metal-core nucleation, providing a bottom-up platform for constructing atomically precise alloy clusters with programmable architectures and tunable optical properties.

## 2 Results and discussion

### 2.1. Synthesis and structural analysis

**Fig. 2:**
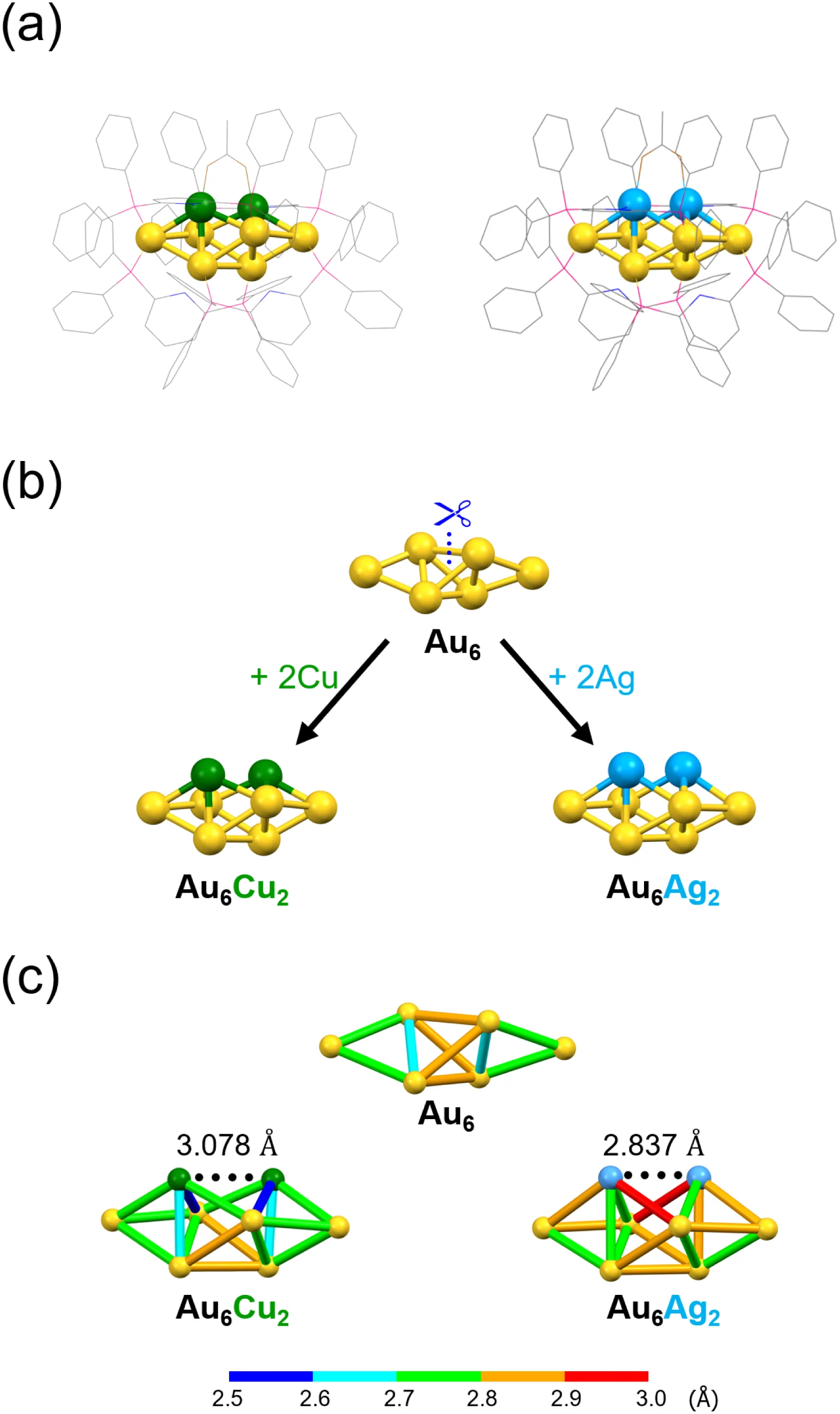
(a) X-ray crystal structures, (b) metal frameworks and (c) bond length heat maps of Au_6_Cu_2_ (left) and Au_6_Ag_2_ (right). In (b) and (c), the framework of Au_6_ is also shown. In (c), black dotted lines indicate the distances between the heterometal atoms.

The alloy Au_6_M_2_ clusters were rationally synthesized by reacting a parent [Au_6_(2,6-PyDP)_4_]^2+^ (Au_6_)^26^ with M(i) complexes (M = Cu, Ag). Electrospray ionization (ESI) mass spectrometry revealed dominant triply charged signals at *m*/*z* ∼1052 and ∼1082 *m*/*z*, which were assigned to [Au_6_Cu_2_(2,6-PyDP)_4_(OAc)]^3+^ (Au_6_Cu_2_) and [Au_6_Ag_2_(2,6-PyDP)_4_(OAc)]^3+^ (Au_6_Ag_2_), respectively (Fig. S1). Single-crystal X-ray analysis revealed the transformed structures (Fig. 2a), in which the central Au–Au bond in Au_6_ undergoes cleavage to integrate acetate-bridged M_2_ dimers, effectively reconstructing the framework into a unique Au_6_M_2_ motif (Fig. 2b). Specifically, the Au⋯Au distance at the cleavage site (indicated by the scissors symbol in Fig. 2b) increased from 2.842–2.871 Å in Au_6_ to 3.712 Å in Au_6_Cu_2_ and 3.996 Å in Au_6_Ag_2_. These markedly elongated Au⋯Au separations provide direct structural evidence for cleavage of this specific Au–Au bond during cluster transformation. Metal–metal bond lengths within the cores were visualized using heat maps (Fig. 2c) and summarized in Table S3. Upon alloying, the average Au–Au bond lengths within the remaining intact gold framework slightly increased from 2.768 Å in Au_6_, reaching 2.783 Å in Au_6_Cu_2_ and 2.796 Å in Au_6_Ag_2_, respectively. Although the changes in Au–Au bonds are marginal upon alloying, the M–Au and M–M distances differ markedly between the two alloy clusters. Reflecting the smaller van der Waals (vdW) radii of Cu (1.40 Å) compared to Ag (1.72 Å), the average M–Au distances are shorter in Au_6_Cu_2_ (2.668 Å) than in Au_6_Ag_2_ (2.840 Å), leading to a more compact core in Au_6_Cu_2_. For the M_2_ dimers, the M–M distances (black dotted lines in Fig. 2c) were 3.078 Å for Au_6_Cu_2_ and 2.837 Å for Au_6_Ag_2_, *i.e.*, ∼0.3 Å longer and ∼0.6 Å shorter than their respective vdW sums (2.80 and 3.44 Å). The substantially short Ag–Ag contacts in Au_6_Ag_2_ may suggest possible Ag–Ag bonding; nevertheless, theoretical analyses do not support the existence of a direct Ag–Ag bond (see below).

**Fig. 3:**
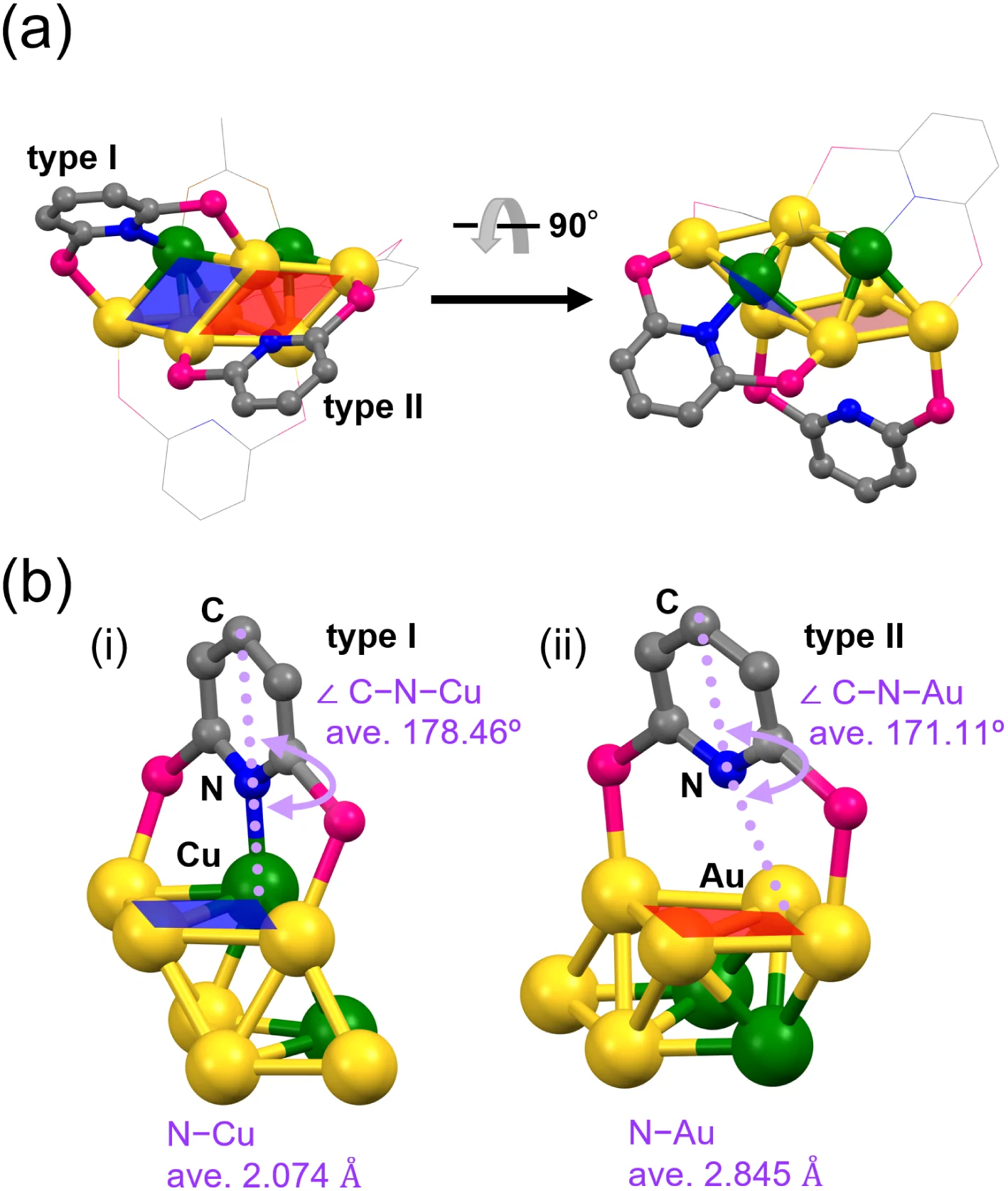
(a) Arrangement of 2,6-PyDP in Au_6_Cu_2_ and (b) two coordination modes: (i) N–Cu bond (type I) and (ii) N–Au interaction (type II).

Comparison of Au_6_ and Au_6_M_2_ highlights the role of the pyridyl-bridged diphosphine ligand in directing heterometal anchoring. In Au_6_, all four pyridyl nitrogen atoms (N) were located away from the gold framework, with the average N–Au distance of 3.582 Å. This distance is significantly longer than the vdW sum of N and Au (3.21 Å), thus leaving the N atoms available for coordination. In contrast, Au_6_M_2_ displayed two N atoms closer to the M atoms (N–M distance <2.4 Å) and the remaining two closer to the Au atoms (N–Au distance <2.9 Å) (Table S4), forming two distinct coordination modes (types I and II; Fig. 3 for Au_6_Cu_2_). In type I (Fig. 3b(i)), a pair of Au–P bonds of 2,6-PyDP exhibited a clear tilt toward the metal framework, and almost a linear alignment was observed between the pyridine ring and Cu atom (average C–N–Cu angle: 178.46°; Table S5), forming a short, dative N → Cu bond (2.074 Å on average). In type II (Fig. 3b(ii)), the longer distances between the pyridine rings and Au atoms suggested weaker interactions. Consequently, the coordination of pyridyl N atoms to the M centers drives the rearrangement of the Au_6_ skeleton upon M-atom incorporation, yielding the unique Au_6_M_2_ framework.

### 2.2. Optical absorptions and electronic structures

**Fig. 4:**
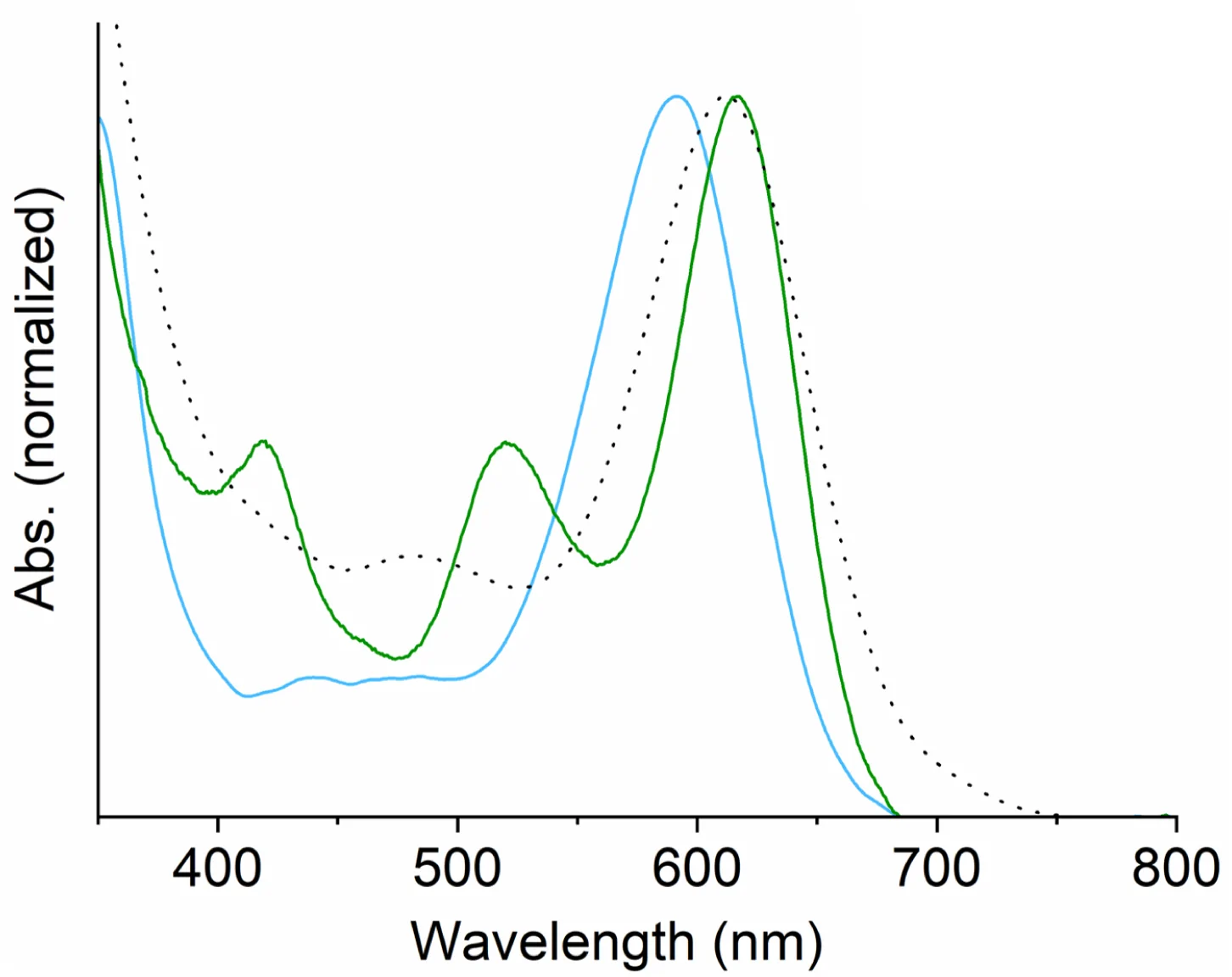
Absorption spectra of Au_6_Cu_2_ (green line) and Au_6_Ag_2_ (aqua line) in CH_2_Cl_2_. The spectrum of Au_6_ is depicted as a black dotted line.

To elucidate the impact of heterometal incorporation on the electronic structures of Au_6_, the UV-vis absorption spectra of Au_6_M_2_ and Au_6_ were measured (Fig. 4). The spectral profiles of the alloy clusters were similar to those of Au_6_. Specifically, Au_6_Cu_2_ and Au_6_Ag_2_ exhibited the longest-wavelength absorption bands at 616 and 592 nm, respectively, representing a 4 nm red-shift and a 20 nm blue-shift relative to Au_6_ (612 nm).

These trends—longer wavelength absorption for Cu-containing clusters compared to Ag-containing clusters, and a blue-shift for Ag-containing clusters relative to pure gold clusters—are consistent with previous reports on gold-based alloy clusters.^27–32^ These observations indicate that alloying perturbs the electronic structure of Au_6_.

**Fig. 5:**
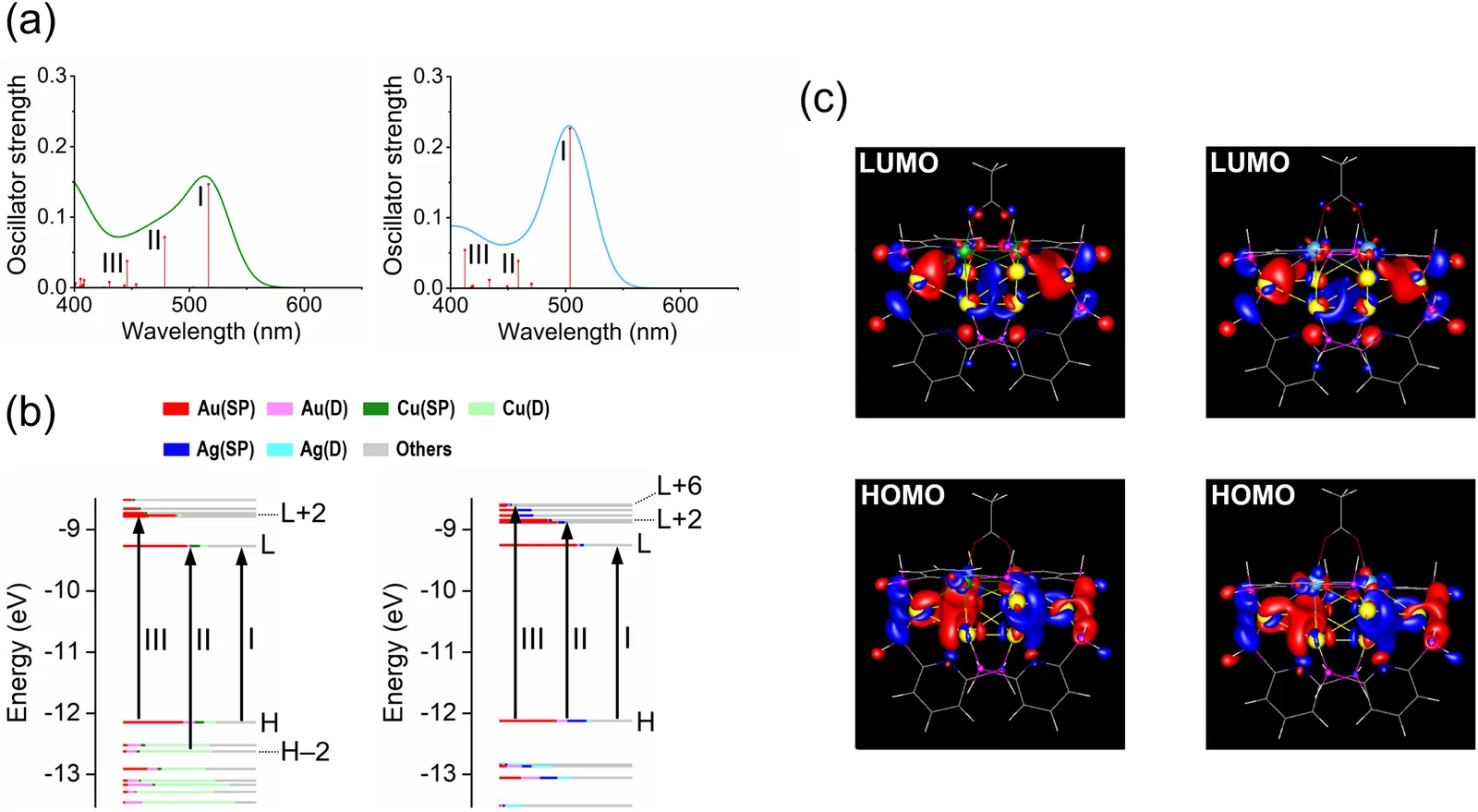
(a) Calculated absorption spectra, (b) energy diagrams and (c) frontier MOs of Au_6_Cu_2_ (left) and Au_6_Ag_2_ (right). In (b), H and L represent HOMO and LUMO, respectively.

The electronic structures of the clusters were analyzed using theoretical calculations on simplified models of Au_6_M_2_, where the phenyl groups of the diphosphines were replaced with hydrogen atoms. The theoretical absorption spectra closely reproduced the experimental spectra, and the longest-wavelength band (transition I) was assigned to the HOMO–LUMO transition in all three clusters (Fig. 5a, b, S4a and b). The frontier orbitals (HOMOs and LUMOs) of Au_6_M_2_ (Fig. 5c) closely resemble those of Au_6_ (Fig. S4c), with dominant Au(6sp) contributions, indicating minimal heterometal involvement in these orbitals and supporting the perturbation picture. We further examined the electronic character of the heterometals in these alloy clusters. Based on the formal charge of M (+1), the heterometal atoms are expected to adopt a monovalent cation-like state, which is consistent with the natural atomic charge of M (approximately +0.6) from natural bond orbital analysis (Fig. S5).

**Fig. 6:**
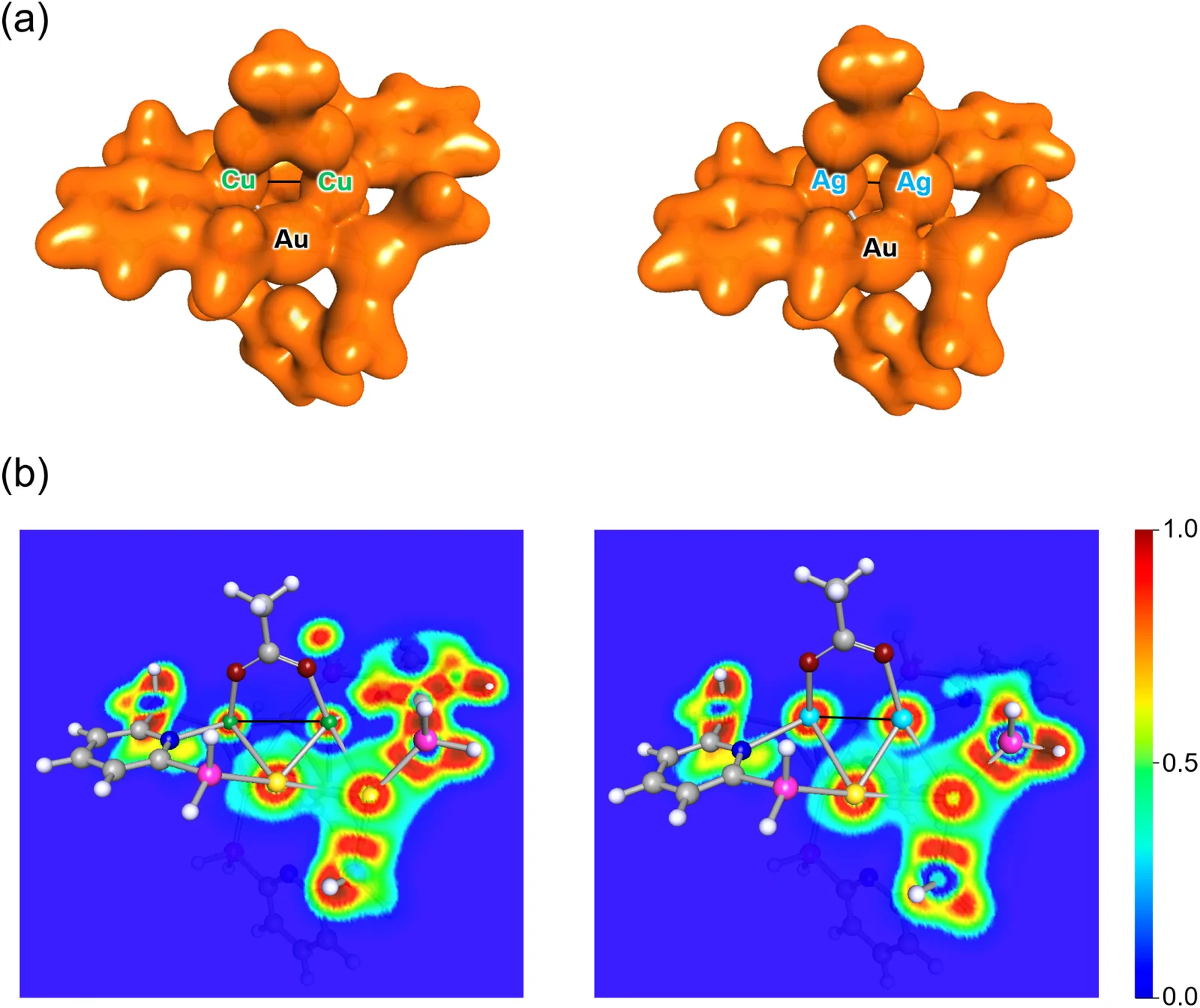
(a) Total electron density surfaces and (b) electron localization function maps of Au_6_Cu_2_ (left) and Au_6_Ag_2_ (right). Cut planes in (b) are defined by triangular M_2_Au units in (a).

To examine the bonding nature of the heterometal species, we calculated the total electron density of Au_6_M_2_. Surprisingly, despite an M–M distance shorter than the vdW sum by ∼0.6 Å, Au_6_Ag_2_ showed no high-density region between the M atoms (Fig. 6a, right, black line). Such a high-density region is typically expected for metallic or covalent bonding along the internuclear axis. To clarify the absence of direct M–M bonding, we subsequently employed the electron localization function (ELF) analysis, which provides insight into metal–metal interactions.^33^ ELF values range from 0 (blue, very low electron density) to 1 (red, perfect localization), with 0.5 (green) representing free-electron-like, delocalized behavior. The ELF maps focusing on the triangular M_2_Au units of both Au_6_M_2_ (Fig. 6b) displayed broad blue regions between the two M atoms, confirming the absence of bonding electron density and thus no direct M–M interaction. Blue regions were also evident at one of the two M–Au contacts, indicating minimal electron density associated with bonding around the M atoms. Collectively, these results demonstrate that the cationic heterometal atoms exhibit negligible intermetallic interactions; instead, their anchoring is predominantly governed by coordination through the pyridyl groups.

### 2.3. Heterometal exchange

Considering the limited M–M and M–Au bonding character indicated by the electron density and ELF analyses, the heterometal species in Au_6_M_2_ appear to be relatively labile, making them potentially replaceable. To probe the heterometal exchange behavior, solutions of Au_6_Ag_2_ and Au_6_Cu_2_ were treated with two equivalents of Cu(i) and Ag(i), respectively. Upon addition of Cu(i), Au_6_Ag_2_ exhibited a red-shift of its longest-wavelength absorption band from 592 to 616 nm, suggesting the formation of Au_6_Cu_2_, whereas the spectrum of Au_6_Cu_2_ remained unchanged upon treatment with Ag(i) (Fig. S6a). ESI mass spectrometry further confirmed the formation of Au_6_Cu_2_ in a solution that initially contained Au_6_Ag_2_ (Fig. S6b, top). These results unambiguously demonstrate selective heterometal exchange: Ag-to-Cu replacement proceeds in Au_6_Ag_2_, whereas Cu-to-Ag replacement does not occur in Au_6_Cu_2_. A plausible interpretation is that Au_6_Cu_2_ is thermodynamically favored over Au_6_Ag_2_ because of the stronger N → M coordination^34^ and more compact metal framework with longer M–M separation, which likely reduces nonbonded M–M repulsion and stabilize the structure. To quantitatively support this interpretation, theoretical calculations were performed. The reaction energy for the Ag-to-Cu exchange process was found to be −215.8 kJ mol^−1^, indicating that the formation of Au_6_Cu_2_ is thermodynamically favored (Table S12). In line with this, Au_6_Cu_2_ exhibited markedly higher chemical stability than Au_6_Ag_2_ in solution, as evidenced by its smaller spectral changes (Fig. S7).

### 2.4. Photoluminescence

**Fig. 7:**
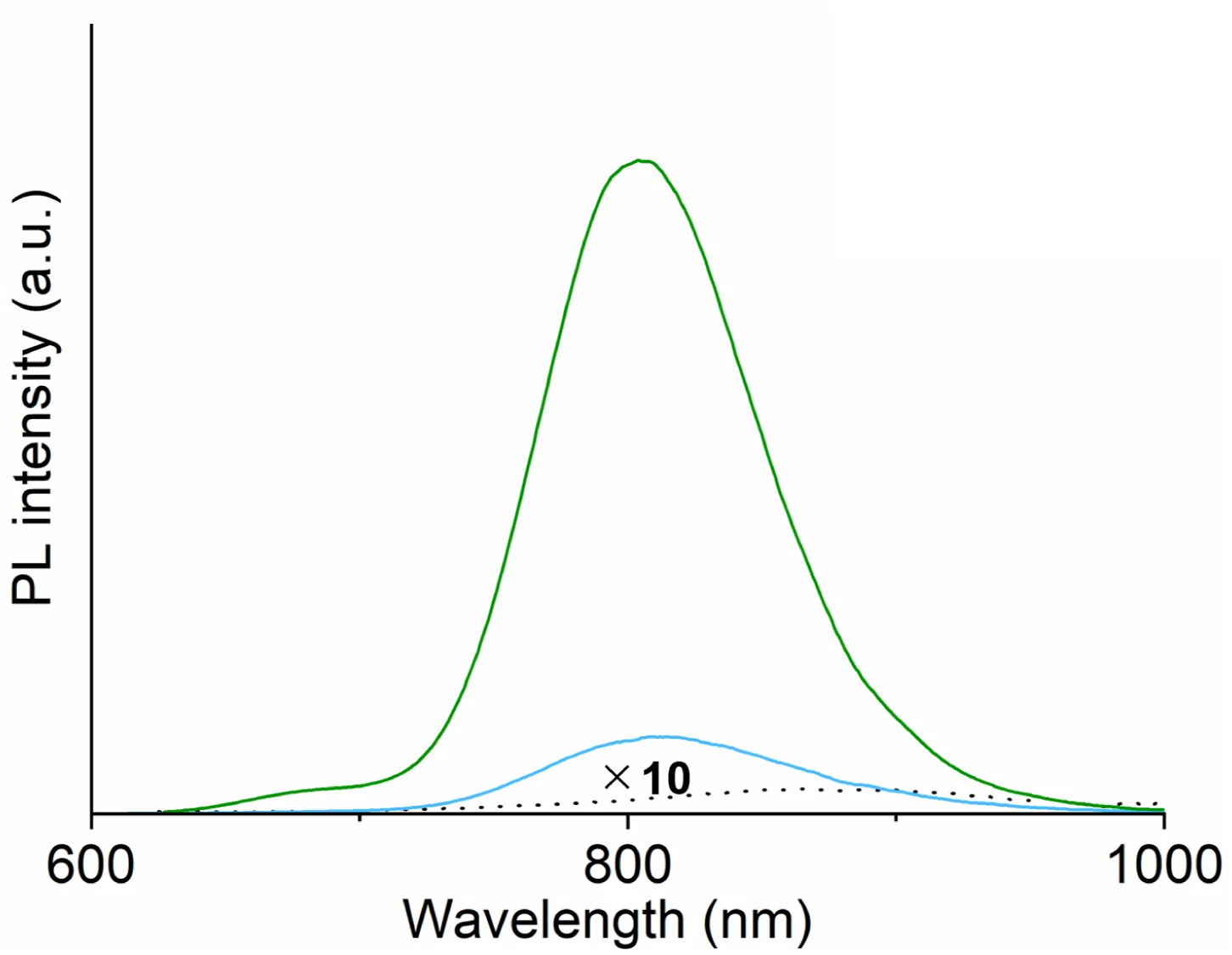
Photoluminescence spectra of Au_6_Cu_2_ (green line) and Au_6_Ag_2_ (aqua line) in CH_2_Cl_2_. The spectrum of Au_6_ is depicted as a black dotted line (scaled by a factor of 10).

To investigate the influence of heterometal incorporation on photophysical properties, we measured the photoluminescence spectra of Au_6_M_2_ and Au_6_ in CH_2_Cl_2_. All clusters exhibited near-infrared (NIR) emission, with Au_6_Cu_2_ and Au_6_Ag_2_ emitting near 800 nm (805 and 810 nm, respectively), whereas Au_6_ showed a pronounced red-shift to 865 nm (Fig. 7). Time-resolved photoluminescence decay profiles (Fig. S8) further revealed microsecond-scale lifetimes of 14.22 µs for Au_6_Cu_2_ and 1.77 µs for Au_6_Ag_2_, characteristic of triplet-state phosphorescence. In contrast, quantum yield (QY) analysis revealed striking differences: Au_6_Cu_2_ reached 29.9%, compared to 4.0% for Au_6_Ag_2_ and only 0.2% for Au_6_. Notably, the QY for Au_6_Cu_2_ is relatively high among the previously reported NIR-emissive gold-based alloy clusters (Table S13). These results highlight that alloying enhances the emissive efficiency, with Cu incorporation providing the most pronounced improvement (150-fold enhancement). A similar luminescence enhancement upon Cu alloying was observed for an alkynyl-protected Au_16_Cu_6_ cluster that exhibits a near-unity quantum yield. In that case, the enhanced emission was attributed to a Cu-induced contraction of the metal framework, which suppressed nonradiative decay and accelerated intersystem crossing.^6^

Taking our structural and computational analyses into account, the markedly higher quantum yield of Au_6_Cu_2_ may be associated with Cu-induced coordination and geometric effects. Specifically, the crystal structures reveal that the Cu–Au and Cu–N distances in Au_6_Cu_2_ are significantly shorter than those of their Ag counterparts by 0.172 and 0.259 Å, respectively (Tables S3 and S4). This contraction originates from the smaller ionic radius of Cu(i) and its stronger affinity for N-donor ligands compared to Ag(i), which is further supported by the theoretical calculations suggesting enhanced thermodynamic stabilization and stronger N → Cu coordination in Au_6_Cu_2_ (Table S12). Consequently, these tighter Cu–N interactions and shorter Cu–Au contacts construct a more constrained and compact cluster framework, which may effectively suppress excited-state structural relaxation and subsequent nonradiative decay. Although further studies are required to elucidate the detailed excited-state dynamics in this system, heterometal anchoring *via* pyridyl-N → M coordination is demonstrated to enhance the emission efficiency of atomically precise gold clusters.

## 3 Conclusions

We have demonstrated a postsynthetic alloying strategy for constructing Au_6_M_2_ (M = Cu, Ag) clusters through the coordination-driven anchoring of M_2_ dimers onto a Au_6_ core. The N-donor sites within the pyridyl-bridged diphosphines dictated the site-specific heterometal grafting, accompanied by a structural rearrangement of the Au_6_ framework, as evidenced by single-crystal X-ray analysis. Intriguingly, the Au_6_Cu_2_ cluster exhibited a distinctly more compact metal core, reflecting shorter M–Au distances compared to its Au_6_Ag_2_ analogue. Furthermore, the exposure of Au_6_Ag_2_ to Cu(i) species triggered a spontaneous Ag-to-Cu exchange, highlighting the superior thermodynamic stability of Au_6_Cu_2_. Despite the modest perturbation of frontier-orbital-based absorption features, heterometal incorporation induced pronounced enhancements in NIR photoluminescence. Most notably, the Au_6_Cu_2_ cluster exhibited a 150-fold increase in quantum yield, underscoring the profound photophysical influence of ligand-anchored heterometals. Theoretical calculations revealed the electron-deficient nature of the heterometal species and their negligible electronic coupling with adjacent metal atoms, consistent with ligand-directed surface anchoring rather than traditional core-level substitution. This work represents a pivotal step toward evolving heterometal alloying from a stochastic outcome into a controllable chemical process, providing a general framework for the rational design of atomically precise alloy clusters with tailored structures and emergent properties.

## Author contributions

Yukatsu Shichibu: writing – original draft, writing – review & editing, conceptualization, methodology, data curation, supervision, formal analysis, project administration, funding acquisition, resources, validation, visualization, investigation. Kazuki Yamada: investigation. Hiroshi Itakura: investigation. Hiroto Morita: investigation. Lan Xu: investigation. Katsuaki Konishi: writing – review & editing, conceptualization, supervision, project administration, funding, acquisition, resources. All authors have approved the final version of the manuscript.

## Conflicts of interest

There are no conflicts to declare.

## Supplementary Material

SC-OLF-D6SC04505G-s001

SC-OLF-D6SC04505G-s002

## Data Availability

CCDC 2540290 and 2540291 contain the supplementary crystallographic data for this paper.^35*a,b*^ The data supporting this article have been included as part of the supplementary information (SI). Supplementary information: experimental procedures, X-ray diffraction data, computational results and further experimental details. See DOI: https://doi.org/10.1039/d6sc04505g.
