# Supplementary material for: N-coordination-driven anchoring of M2 dimers (M = Cu, Ag) on Au6 cluster: structural evolution and enhanced photoluminescence
Source: Chem Sci. 2026 Aug 5;17(35):16763–8. doi: 10.1039/d6sc04505g (PMC13470586; doi:10.1039/d6sc04505g)
Supplement: Supplementary file 1 [file SC-017-D6SC04505G-s001.pdf]

Supplementary Information (SI) for Chemical Science.
This journal is © The Royal Society of Chemistry 2026

Supporting Information

N-Coordination-Driven Anchoring of M_2 Dimers (M = Cu, Ag) on Au_6 Cluster: Structural Evolution and Enhanced Photoluminescence

Yukatsu Shichibu,*[ab] Kazuki Yamada,[a] Hiroshi Itakura,[a] Hiroto Morita,[a] Lan Xu,[a] and Katsuaki Konishi*[ab]

[a] Graduate School of Environmental Science, Hokkaido University, North 10 West 5, Sapporo 060-0810, Japan.
[b] Faculty of Environmental Earth Science, Hokkaido University, North 10 West 5, Sapporo 060-0810, Japan.

E-mail: shichibu@ees.hokudai.ac.jp; konishi@ees.hokudai.ac.jp

Table of Contents

1. *Materials and Methods* 2
   Materials 2
   Synthetic Procedures 2
   Instrumentation 2
   Computational Details 2
2. *Experimental and Computational Results* 3
   ESI Mass Spectra 3
   Crystal Data 4
   ORTEP Drawing 6
   Interatomic Distances and Angles 7
   Electronic Structure Analysis 8
   Atomic Charges 8
   Molecular-Orbital and Electron-Transition Data 9
   Heterometal Exchange 12
   Stability Comparison 13
   Photoluminescence Decay 13
   Comparison of NIR-Emissive Metal Clusters 14
3. *References* 15

1. Materials and Methods

Materials

Acetic acid (95%), potassium hexafluorophosphate (95%) and silver acetate (97%) were obtained from Nacalai Tesque, TCI and Fujifilm Wako, respectively. Solvents were purchased from Kanto Chemicals. All the reagents were used as received. Chloro(triphenylphosphine)copper and $[Au_6(2,6\text{-PyDP})_4](NO_3)_2$ were prepared from $CuCl_2$ and $[Au_9(PPh_3)_8](NO_3)_2$, respectively, according to reported procedures.[1-2]

Synthetic Procedures

$[Au_6Cu_2(2,6\text{-PyDP})_4(OAc)](PF_6)_3$ (**Au_6Cu_2**$\cdot(PF_6)_3$): A methanol solution (17.5 mL) of $[Au_6(2,6\text{-PyDP})_4](NO_3)_2$ (25.0 mg, 8.08 μmol) was mixed with a dichloromethane solution (7.5 mL) of chloro(triphenylphosphine)copper (7.3 mg, 20.2 μmol, 2.5 eq.) and stirred at room temperature for 4 h. After solvent removal, the residue was dissolved in methanol (5 ml) and filtered. A 100 mM acetic acid/methanol solution (161 μl, 16.1 μmol, 2 eq.) was then added, and the mixture was left to stand for 6 h. The solvent was removed by dryness, and the residue was dissolved in a small amount of dichloromethane and reprecipitated in diethyl ether to remove excess acid. The resulting precipitate was collected by centrifugation, dissolved in methanol (5 ml), and treated with potassium hexafluorophosphate (14.9 mg, 80.8 μmol). After stirring for 30 min, the solvent was removed by dryness, and the residue was dissolved in a small amount of dichloromethane, filtered, and reprecipitated in diethyl ether. The final product was washed with diethyl ether and dried to yield a blue powder (11.4 mg, 39.2% based on Au). Crystals suitable for X-ray analysis were grown from dichloromethane/benzene (1:3).

$[Au_6Ag_2(2,6\text{-PyDP})_4(OAc)](PF_6)_3$ (**Au_6Ag_2**$\cdot(PF_6)_3$): A methanol solution (12.5 mL) of $[Au_6(2,6\text{-PyDP})_4](NO_3)_2$ (25.0 mg, 8.08 μmol) was mixed with an aqueous solution (6.25 mL) of silver acetate (3.4 mg, 20.2 μmol, 2.5 eq.) and stirred at room temperature for 24 h. After solvent removal, the residue was dissolved in methanol (5 ml) and filtered. The filtrate was treated with KPF_6 (14.9 mg, 80.8 μmol). After stirring for 30 min, the solvent was removed by dryness, and the residue was dissolved in a small amount of dichloromethane, filtered, and reprecipitated in diethyl ether. The final product was washed with diethyl ether and dried to yield a purple powder (12.4 mg, 41.7% based on Au). Crystals suitable for X-ray analysis were grown from dichloromethane/benzene (1:3).

Instrumentation

Electrospray ionization (ESI) mass spectra were obtained using a high-resolution Bruker micrOTOF-HS mass spectrometer. Optical absorption spectra were recorded using a JASCO V-670 spectrometer. Photoluminescence spectra were measured using a JASCO FP-8600 spectrometer, and the absolute quantum yields were determined relative to rhodamine 6G in ethanol. Photoluminescence lifetimes were determined using a Hamamatsu Photonics Quantaurus-Tau C11367-22 spectrometer with an excitation wavelength of 280 nm. Crystal data were collected on Rigaku MicroMax-007HF diffractometers, employing either Cu Kα radiation (λ = 1.54184 Å) or Mo Kα radiation (λ = 0.71073 Å). Data reduction, scaling and absorption correction were performed using CrysAlisPro software.[3] All crystal structures were solved by intrinsic phasing with SHELXT-2018[4] and refined by full-matrix least-squares on F^2 with SHELXL-2018[5] using Olex2 software.[6] Non-hydrogen atoms of the metal clusters and counter anions were refined anisotropically. The solvent molecules and hydrogen atoms were refined isotropically.

Computational Details

All calculations were performed by density functional theory (DFT) or time-dependent DFT (TDDFT) methods with the B3LYP functional[7-8] and the def2-TZVP basis set[9] with D4 dispersion correction,[10] using TURBOMOLE 7.6 software.[11] Crystallographically determined structures of metal clusters after replacement of the phenyl groups with hydrogen atoms were adopted as initial geometries for the geometry-optimization calculations. The default 60-electron relativistic effective core potential[12] was employed for the gold atoms, and the resolution of identity approximation for Coulomb interactions[13] was used to accelerate the calculations. The amounts of the atomic charges were estimated using natural bond orbital analysis.[14]

2. *Experimental and Computational Results*

ESI Mass Spectra

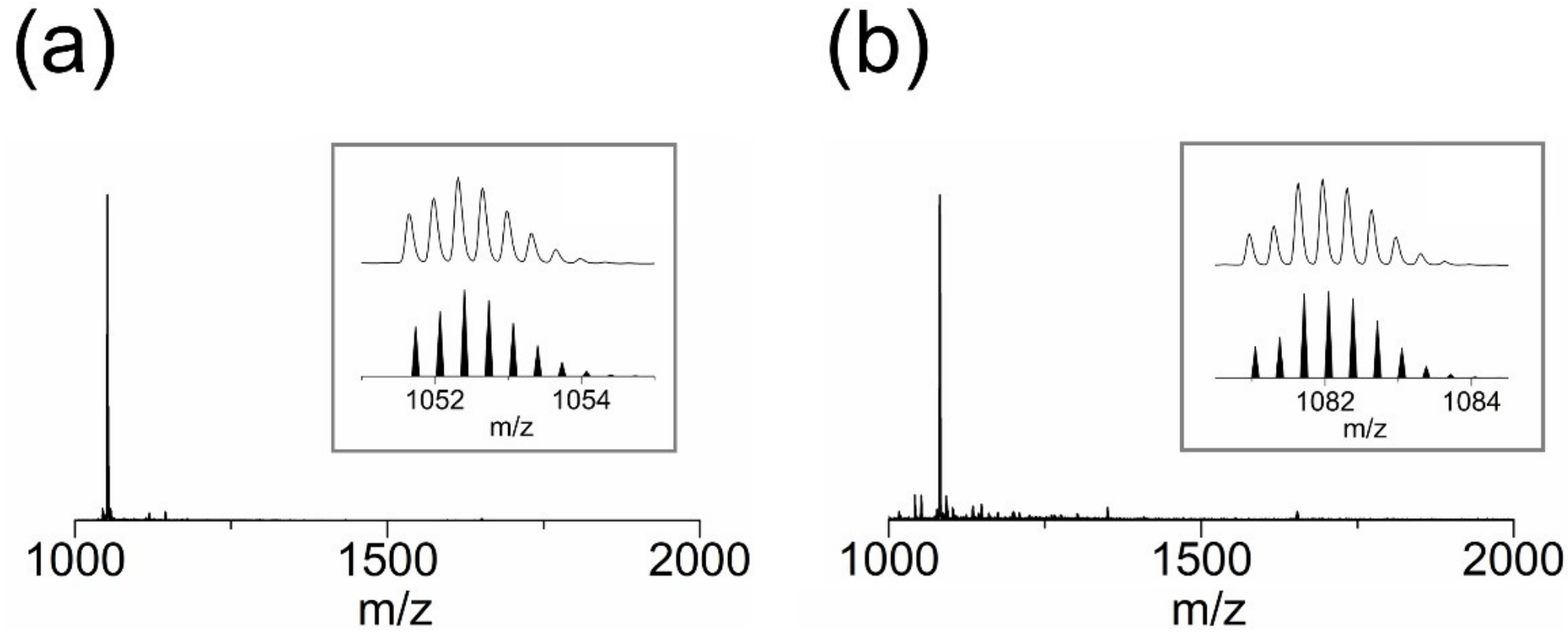

Figure S1. Positive-ion ESI mass spectra of (a) $\mathbf{Au_6Cu_2}$ and (b) $\mathbf{Au_6Ag_2}$. The insets show comparisons of the experimental data (top) with the calculated isotope patterns (bottom).

Crystal Data

Table S1. Crystal data and structure refinement for **Au_6Cu_2**·3(PF_6)·5.5(C_6H_6).

Empirical formula	$C_{151}H_{128}Au_6Cu_2F_{18}N_4O_2P_{11}$	
Formula weight	4022.12 g/mol	
Crystal system	Triclinic	
Space group	$P\bar{1}$	
Unit cell dimensions	a = 17.4507(4) Å	α = 92.110(2)°
	b = 19.1200(5) Å	β = 93.329(2)°
	c = 21.5224(4) Å	γ = 97.361(2)°
Volume	7102.8(3) $Å^3$	
Z	2	
Density (calculated)	1.881 g/cm^3	
Absorption coefficient	13.486 mm^{-1}	
F(000)	3874	
Crystal size	0.16 × 0.02 × 0.01 mm^3	
Radiation	Cu Kα (λ = 1.54184 Å)	
2θ range for data collection	6.084 to 134.156°	
Index ranges	$-20 \le h \le 20$, $-22 \le k \le 22$, $-25 \le l \le 25$	
Reflections collected	82691	
Independent reflections	24866 [R(int) = 0.0463]	
Data / restraints / parameters	24866 / 1731 / 1283	
Goodness-of-fit on F^2	1.030	
Final R indexes [$I \ge 2\sigma(I)$]	R_1 = 0.0780, wR_2 = 0.2037	
Final R indexes (all data)	R_1 = 0.1194, wR_2 = 0.2352	
Largest diff. peak and hole	3.112 and –1.557 $eÅ^{-3}$	

Table S2. Crystal data and structure refinement for $\mathbf{Au_6Ag_2}\cdot3(PF_6)\cdot5.5(C_6H_6)$.

Empirical formula	$C_{151}H_{128}Au_6Ag_2F_{18}N_4O_2P_{11}$	
Formula weight	4110.78 g/mol	
Crystal system	Triclinic	
Space group	$P\bar{1}$	
Unit cell dimensions	a = 17.5679(5) Å	α = 91.423(2)°
	b = 19.1038(5) Å	β = 93.108(2)°
	c = 21.6429(5) Å	γ = 97.160(2)°
Volume	7192.7(3) $Å^3$	
Z	2	
Density (calculated)	1.898 g/cm^3	
Absorption coefficient	6.560 mm^{-1}	
F(000)	3946	
Crystal size	0.28 × 0.10 × 0.09 mm^3	
Radiation	Mo Kα (λ = 0.71073 Å)	
2θ range for data collection	7.112 to 50.246°	
Index ranges	$-20 \le h \le 20$, $-22 \le k \le 22$, $-25 \le l \le 25$	
Reflections collected	150501	
Independent reflections	25569 [R(int) = 0.0463]	
Data / restraints / parameters	25569 / 219 / 1283	
Goodness-of-fit on F^2	1.061	
Final R indexes [$I \ge 2\sigma(I)$]	R_1 = 0.0411, wR_2 = 0.0965	
Final R indexes (all data)	R_1 = 0.0681, wR_2 = 0.1176	
Largest diff. peak and hole	2.739 and –1.413 eÅ^{-3}	

ORTEP Drawing

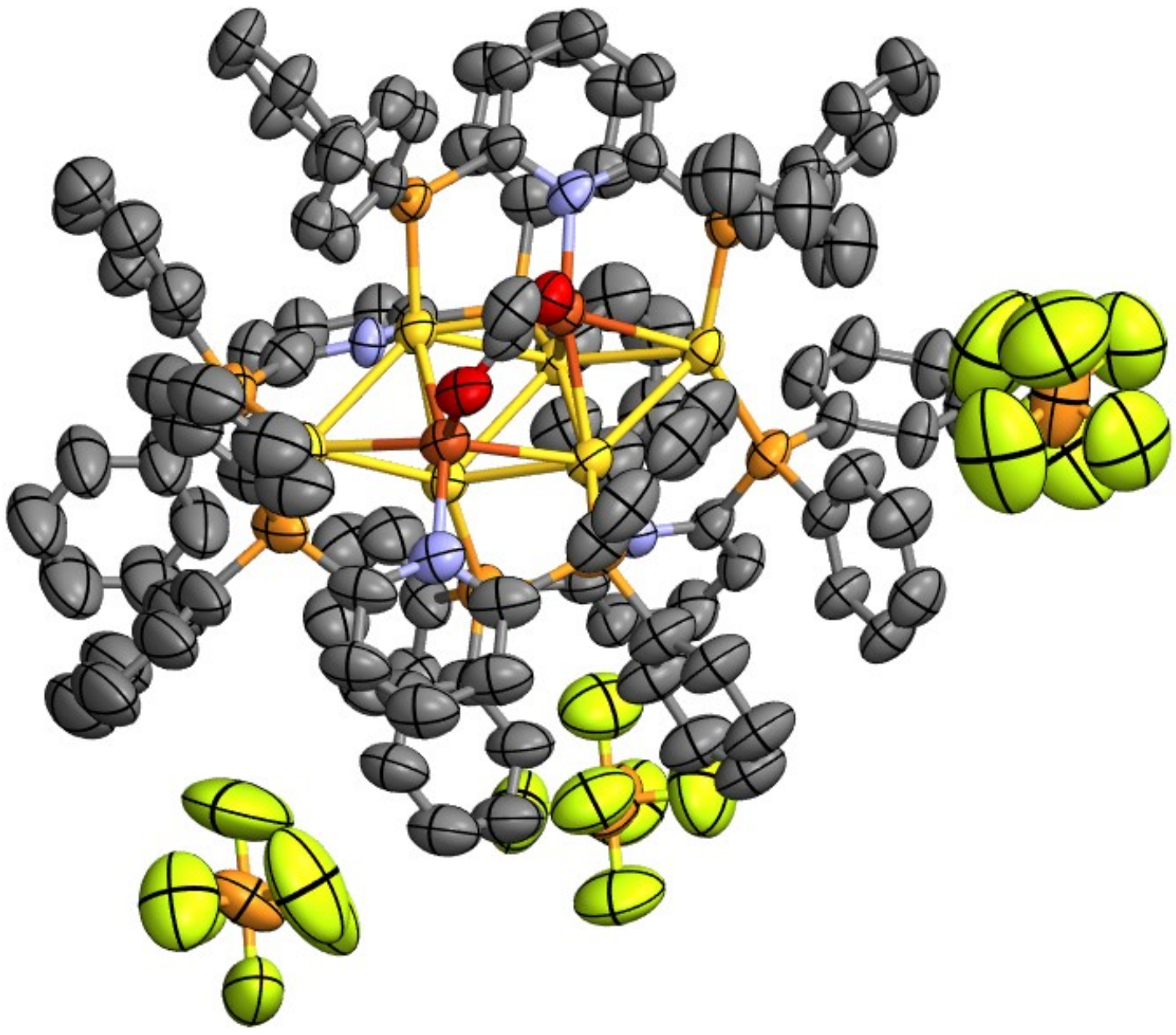

Figure S2. ORTEP drawing for $\mathbf{Au_6Cu_2}\cdot 3(PF_6)\cdot 5.5(C_6H_6)$. Thermal ellipsoids are drawn at the 50% probability level, with solvent molecules and hydrogen atoms omitted for clarity.

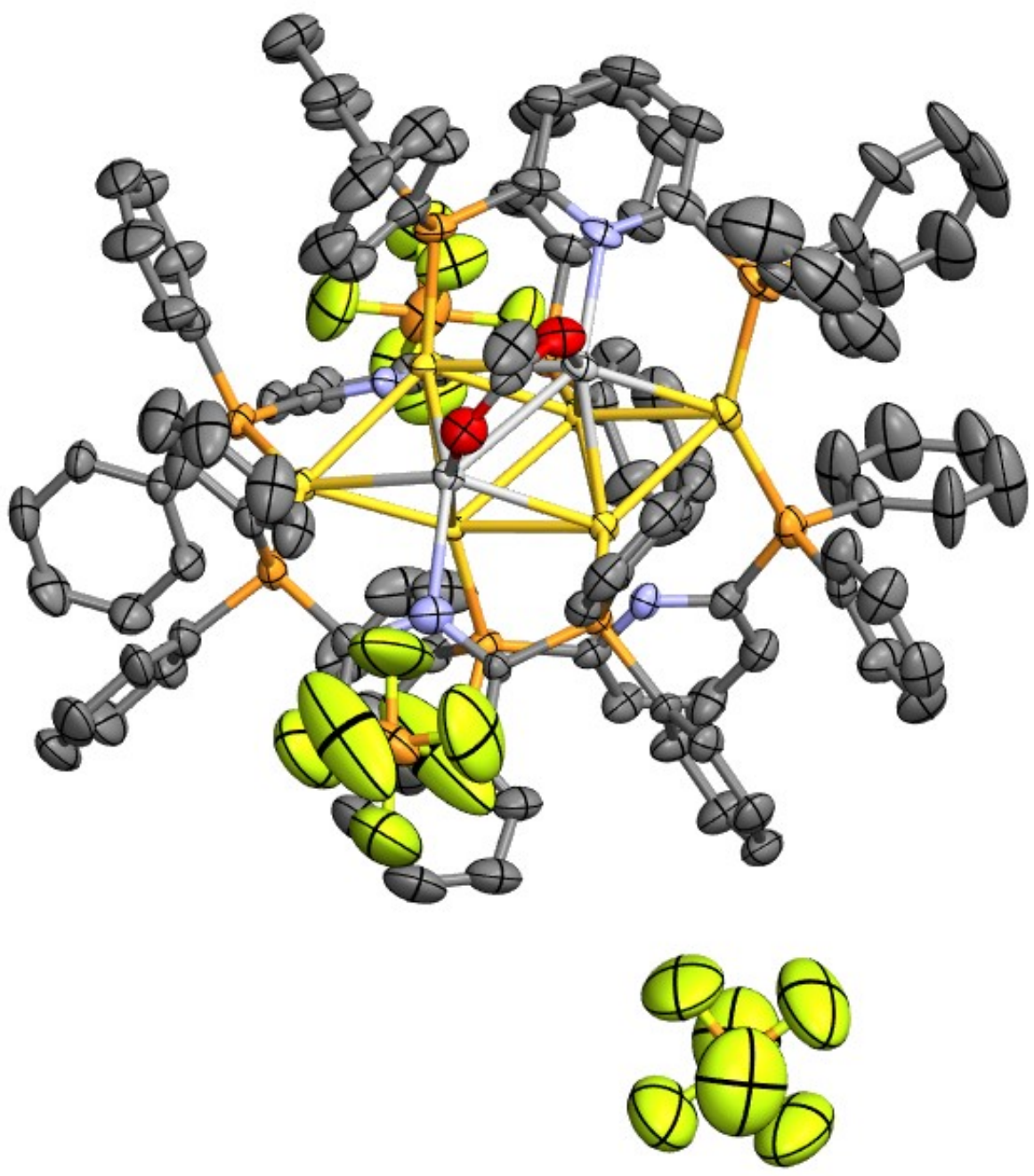

Figure S3. ORTEP drawing for $\mathbf{Au_6Ag_2}\cdot 3(PF_6)\cdot 5.5(C_6H_6)$. Thermal ellipsoids are drawn at the 50% probability level, with solvent molecules and hydrogen atoms omitted for clarity.

Interatomic Distances and Angles

Table S3. Average Au–Au and M–Au bond lengths (Å) of $\mathbf{Au_6}$, $\mathbf{Au_6Cu_2}$ and $\mathbf{Au_6Ag_2}$.[a]

	$\mathbf{Au_6}$	$\mathbf{Au_6Cu_2}$	$\mathbf{Au_6Ag_2}$
Au–Au	2.768	2.783	2.796
	[2.620, 2.871]	[2.705, 2.864]	[2.725, 2.878]
M–Au	–	2.668	2.840
		[2.581, 2.731]	[2.748, 2.939]

[a] Minimum and maximum values are given in square brackets.

Table S4. Average N–M and N–Au distances (Å) of $\mathbf{Au_6}$, $\mathbf{Au_6Cu_2}$ and $\mathbf{Au_6Ag_2}$.[a,b]

	$\mathbf{Au_6}$	$\mathbf{Au_6Cu_2}$	$\mathbf{Au_6Ag_2}$
N–M	–	2.074	2.333
(type I)		[2.073, 2.074]	[2.329, 2.337]
N–Au	3.582	2.845	2.660
(type II)	[3.492, 3.678]	[2.836, 2.854]	[2.640, 2.679]

[a] Minimum and maximum values are given in square brackets. [b] Types I and II coordination modes of 2,6-PyDP in $\mathbf{Au_6M_2}$ are shown in Figure 3, and $\mathbf{Au_6}$ without M appears only for type II.

Table S5. Average C–N–M and C–N–Au angles (°) of $\mathbf{Au_6}$, $\mathbf{Au_6Cu_2}$ and $\mathbf{Au_6Ag_2}$.[a,b]

	$\mathbf{Au_6}$	$\mathbf{Au_6Cu_2}$	$\mathbf{Au_6Ag_2}$
∠ C−N−M	–	178.46	175.98
(type I)		[178.32, 178.59]	[175.51, 176.44]
∠ C−N−Au	169.43	171.11	170.21
(type II)	[168.86, 170.00]	[169.67, 172.55]	[167.99, 172.42]

[a] Minimum and maximum values are given in square brackets. [b] Types I and II coordination modes of 2,6-PyDP in $\mathbf{Au_6M_2}$ are shown in Figure 3, and $\mathbf{Au_6}$ without M appears only for type II. The carbon atom at the para position of the pyridyl group is denoted as C.

Electronic Structure Analysis

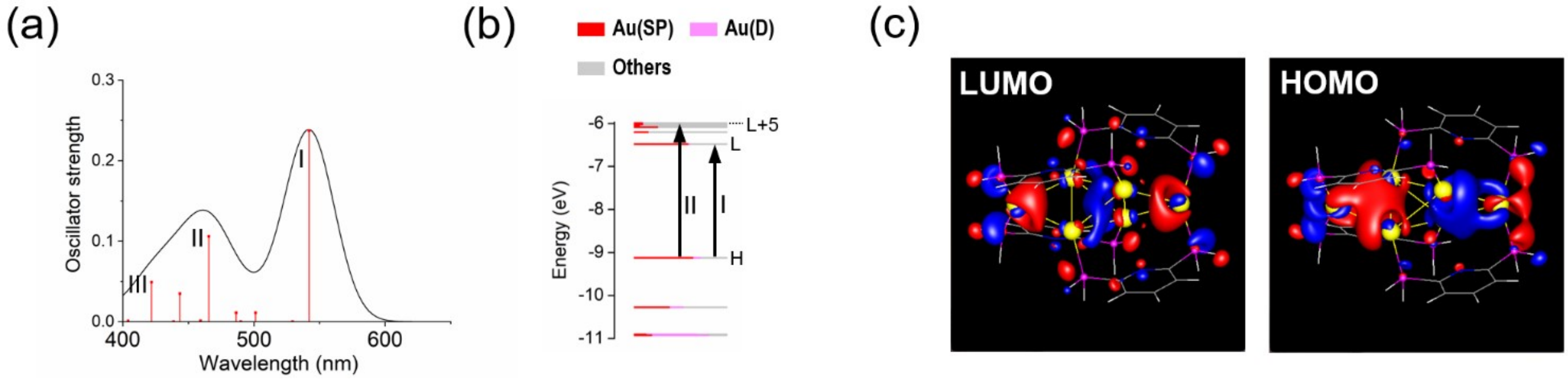

Figure S4. (a) Calculated absorption spectrum, (b) energy diagram and (c) frontier MOs of **Au_6**. In (b), H and L represent HOMO and LUMO, respectively.

Atomic Charges

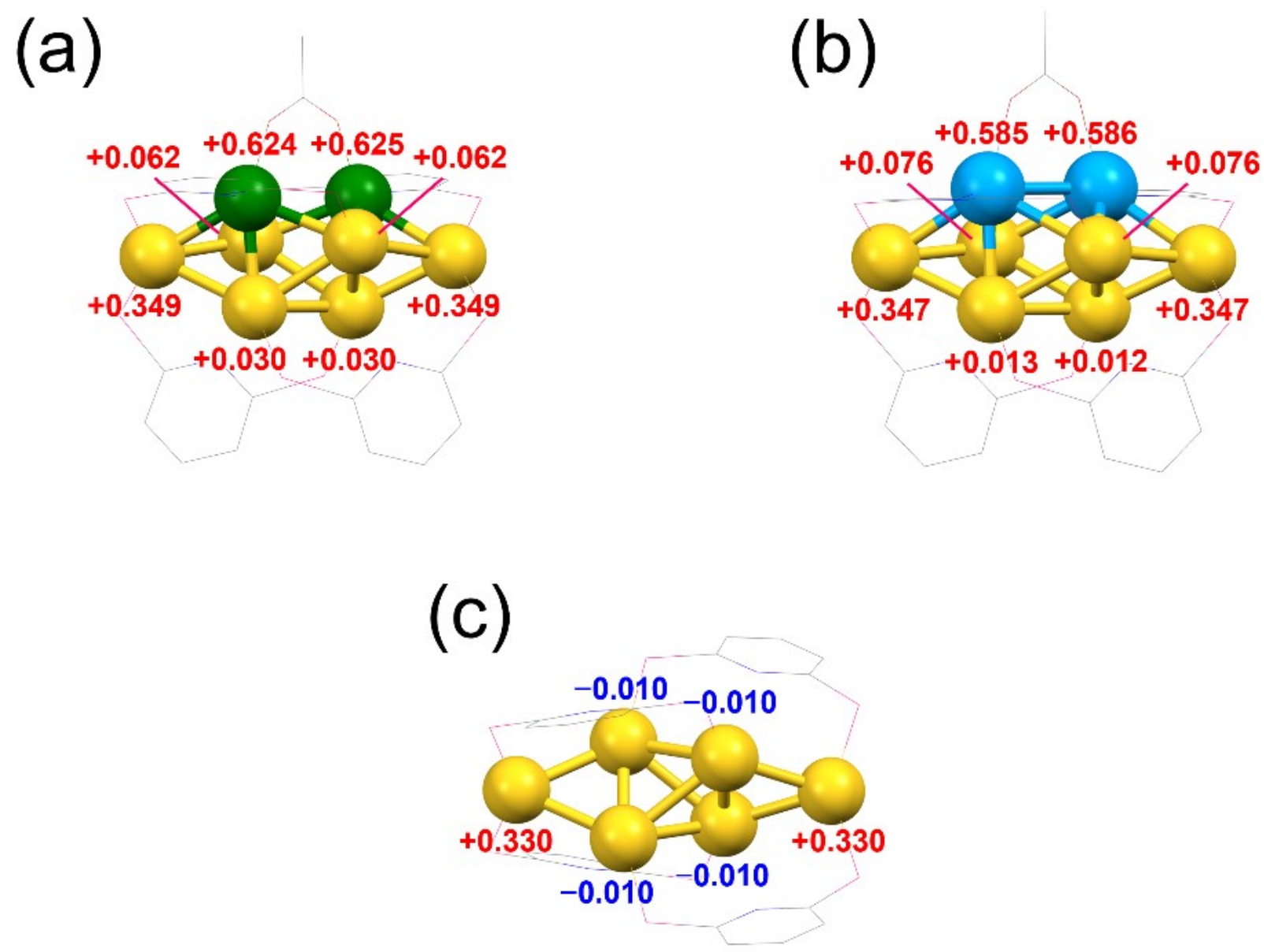

Figure S5. Natural atomic charges of metal atoms in (a) **Au_6Cu_2**, (b) **Au_6Ag_2** and (c) **Au_6**.

Molecular-Orbital and Electron-Transition Data

Table S6. Kohn-Sham orbitals, energies and atomic orbital contributions of **Au_6Cu_2**.

MO	Orbital index	Orbital energy (eV)	% Au (SP)	% Au (D)	% Cu (SP)	% Cu (D)
LUMO+6	255a	–8.51	5	0	2	1
LUMO+5	254a	–8.52	6	0	2	0
LUMO+4	253a	–8.66	11	0	1	2
LUMO+3	252a	–8.73	15	0	2	2
LUMO+2	251a	–8.77	39	1	0	4
LUMO+1	250a	–8.79	17	1	1	6
LUMO	249a	–9.27	48	2	7	6
HOMO	248a	–12.14	45	9	7	10
HOMO–1	247a	–12.52	3	10	3	48
HOMO–2	246a	–12.62	2	8	2	55
HOMO–3	245a	–12.91	18	8	3	34
HOMO–4	244a	–13.10	3	9	1	56
HOMO–5	243a	–13.17	3	19	2	57
HOMO–6	242a	–13.29	3	6	1	59
HOMO–7	241a	–13.46	1	13	0	70

Table S7. Excited states, wavelengths, oscillator strengths and primary orbital-orbital transitions of **Au_6Cu_2**.

Excitation index	Wavelength (nm)	Oscillator strength	Dominant transition	Nature of transition [a]
1	517	1.47×10^{-1}	248a→249a	H→L
2	504	1.00×10^{-4}	247a→249a	H–1→L
3	478	7.16×10^{-2}	246a→249a	H–2→L
4	454	4.64×10^{-3}	248a→250a	H→L+1
5	446	3.77×10^{-2}	248a→251a	H→L+2
6	443	3.11×10^{-3}	248a→252a	H→L+3
7	430	7.97×10^{-3}	248a→253a	H→L+4
8	426	4.37×10^{-5}	245a→249a	H–3→L
9	408	1.05×10^{-2}	247a→252a	H–1→L+3
10	408	4.38×10^{-3}	247a→250a, 244a→249a	H–1→L+1, H–4→L
11	407	2.60×10^{-3}	248a→255a, 247a→253a, 246a→252a	H→L+6, H–1→L+4, H–2→L+3
12	405	1.23×10^{-2}	248a→254a, 244a→249a	H→L+5, H–4→L
13	405	1.04×10^{-3}	247a→251a, 248a→255a, 246a→250a	H–1→L+2, H→L+6, H–2→L+1
14	401	6.34×10^{-3}	243a→249a	H–5→L

[a] H and L represent HOMO and LUMO, respectively.

Table S8. Kohn-Sham orbitals, energies and atomic orbital contributions of $\mathbf{Au_6Ag_2}$.

MO	Orbital index	Orbital energy (eV)	% Au (SP)	% Au (D)	% Ag (SP)	% Ag (D)
LUMO+6	245a	–8.59	7	1	1	0
LUMO+5	244a	–8.61	3	0	2	0
LUMO+4	243a	–8.68	12	1	11	0
LUMO+3	242a	–8.77	14	0	11	1
LUMO+2	241a	–8.85	36	1	2	1
LUMO+1	240a	–8.88	43	1	5	1
LUMO	239a	–9.25	58	2	3	2
HOMO	238a	–12.12	43	8	14	4
HOMO–1	237a	–12.84	1	2	2	16
HOMO–2	236a	–12.87	5	11	7	16
HOMO–3	235a	–13.06	16	14	13	10
HOMO–4	234a	–13.51	0	2	2	13

Table S9. Excited states, wavelengths, oscillator strengths and primary orbital-orbital transitions of $\mathbf{Au_6Ag_2}$.

Excitation index	Wavelength (nm)	Oscillator strength	Dominant transition	Nature of transition [a]
1	504	2.27×10^{-1}	238a→239a	H→L
2	470	6.34×10^{-3}	238a→240a	H→L+1
3	459	3.87×10^{-2}	238a→241a	H→L+2
4	449	2.32×10^{-3}	238a→242a	H→L+3
5	434	1.19×10^{-2}	238a→243a	H→L+4
6	419	3.28×10^{-3}	238a→244a, 236a→239a	H→L+5, H–2→L
7	419	2.03×10^{-3}	237a→239a	H–1→L
8	418	1.53×10^{-3}	236a→239a, 238a→244a	H–2→L, H→L+5
9	412	5.42×10^{-2}	238a→245a	H→L+6

[a] H and L represent HOMO and LUMO, respectively.

Table S10. Kohn-Sham orbitals, energies and atomic orbital contributions of **Au_6**.

MO	Orbital index	Orbital energy (eV)	% Au (SP)	% Au (D)
LUMO+10	215a	–5.41	51	4
LUMO+9	214a	–5.66	30	1
LUMO+8	213a	–5.78	2	1
LUMO+7	212a	–5.81	3	1
LUMO+6	211a	–5.81	33	0
LUMO+5	210a	–5.99	9	1
LUMO+4	209a	–6.02	9	1
LUMO+3	208a	–6.05	6	0
LUMO+2	207a	–6.09	25	0
LUMO+1	206a	–6.20	15	0
LUMO	205a	–6.48	58	2
HOMO	204a	–9.12	63	8
HOMO–1	203a	–10.28	38	15
HOMO–2	202a	–10.91	13	54
HOMO–3	201a	–10.93	19	61

Table S11. Excited states, wavelengths, oscillator strengths and primary orbital-orbital transitions of **Au_6**.

Excitation index	Wavelength (nm)	Oscillator strength	Dominant transition	Nature of transition [a]
1	542	2.37×10^{-1}	204a→205a	H→L
2	529	8.88×10^{-5}	204a→206a	H→L+1
3	501	1.12×10^{-2}	204a→207a	H→L+2
4	490	2.89×10^{-7}	204a→208a	H→L+3
5	486	1.12×10^{-2}	204a→209a	H→L+4
6	466	1.06×10^{-1}	204a→210a	H→L+5
7	459	1.04×10^{-3}	204a→211a	H→L+6
8	444	3.50×10^{-2}	204a→212a	H→L+7
9	439	2.87×10^{-6}	204a→213a	H→L+8
10	422	4.91×10^{-2}	204a→214a	H→L+9
11	404	9.26×10^{-4}	204a→215a	H→L+10

[a] H and L represent HOMO and LUMO, respectively.

Heterometal Exchange

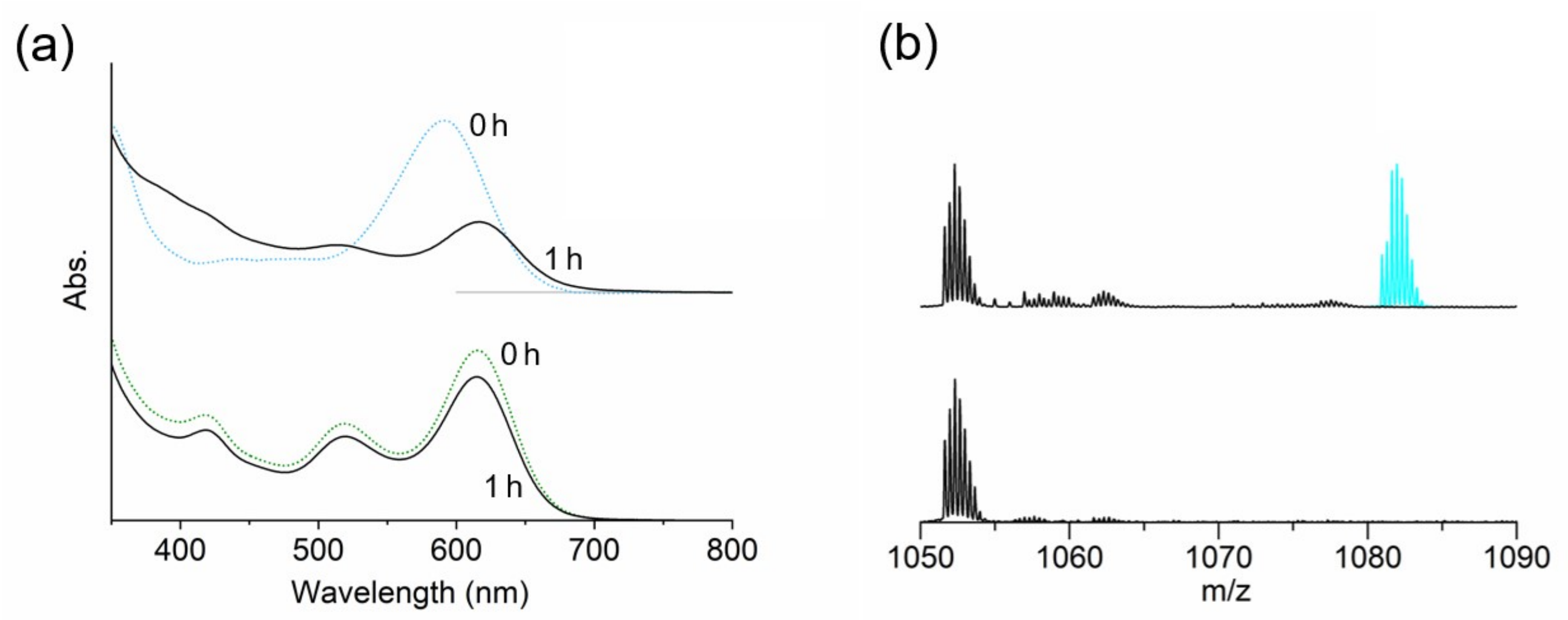

Figure S6. (a) Absorption spectra and (b) positive-ion ESI mass spectra of treatments of $\mathbf{Au_6Ag_2}$ with Cu(I) ion (top) and $\mathbf{Au_6Cu_2}$ with Ag(I) ion (bottom) for 1 h in CH_2Cl_2. In (b), ESI mass spectrum of $\mathbf{Au_6Ag_2}$ is shown overlaid in aqua.

Table S12. Calculated total energies (E) and the reaction energy (ΔE) for the heterometal exchange process.

Species	E (Hartree)
$\mathbf{Au_6Cu_2}$ [a]	–8051.93403
$\mathbf{Au_6Ag_2}$ [a]	–5064.92204
Cu^+	–1640.13460
Ag^+	–146.66970
Reaction	ΔE (Hartree / kJ mol^{-1}) [b]
$\mathbf{Au_6Ag_2}$ + 2 Cu^+ → $\mathbf{Au_6Cu_2}$ + 2 Ag^+	–0.08219 / –215.8

[a] Simplified models of $\mathbf{Au_6M_2}$, where the phenyl groups of the diphosphine ligands were replaced with hydrogen atoms.
[b] Defined as: $\Delta E = [E(\mathbf{Au_6Cu_2}) + 2\,E(Ag^+)] - [E(\mathbf{Au_6Ag_2}) + 2\,E(Cu^+)]$.

Stability Comparison

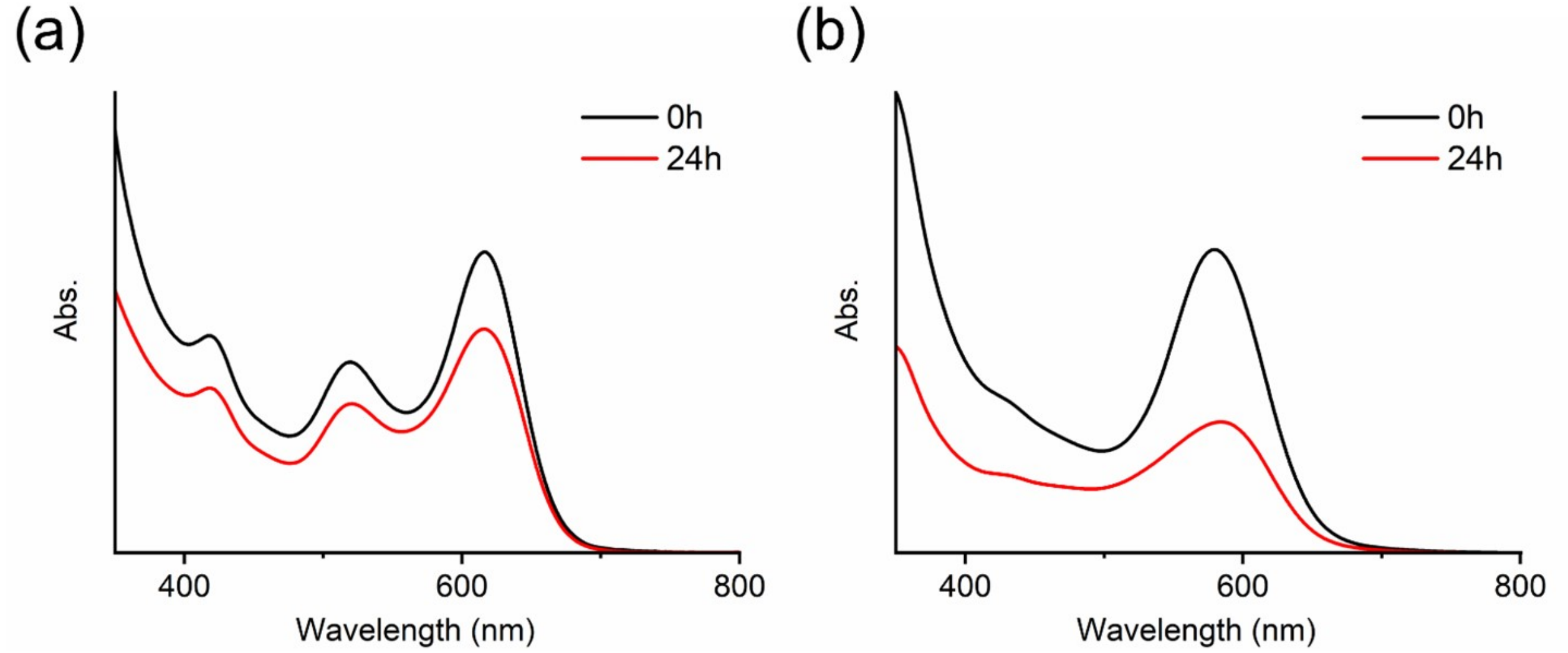

Figure S7. Absorption spectra of (a) $\mathbf{Au_6Cu_2}$ and (b) $\mathbf{Au_6Ag_2}$ in CH_2Cl_2 at 0 h and 24 h.

Photoluminescence Decay

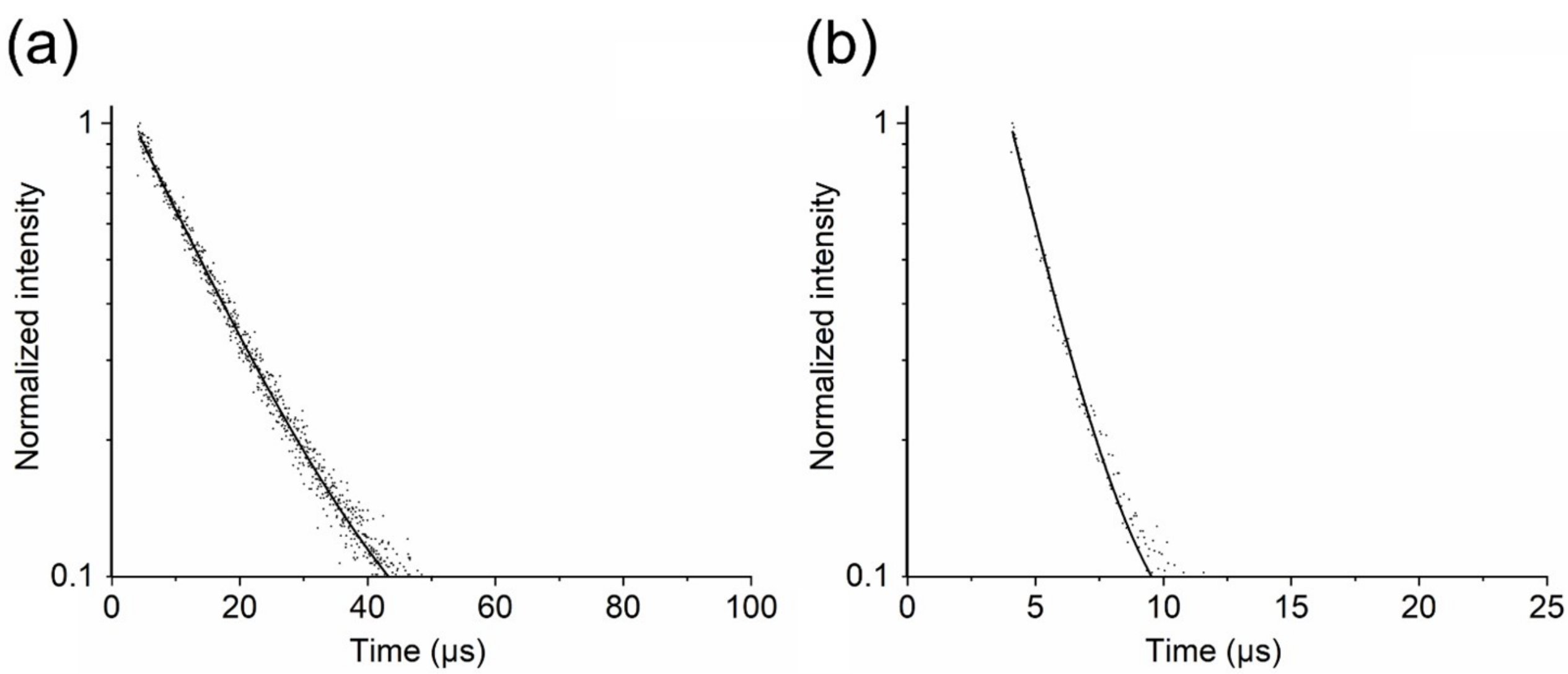

Figure S8. Photoluminescence decay profiles of (a) $\mathbf{Au_6Cu_2}$ (monitored at 805 nm) and (b) $\mathbf{Au_6Ag_2}$ (monitored at 809 nm) in CH_2Cl_2 with excitation at 280 nm. The solid lines indicate the fitting results.

Comparison of NIR-Emissive Metal Clusters

Table S13. NIR photoluminescence properties of **Au_6M_2** and other Au–Ag and Au–Cu alloy clusters in solution.

Cluster	Metal nuclearity	Emission maximum (nm)	QY (%)	Ref.
Au_6Cu_2	8	805	29.9	*this work*
Au_6Ag_2	8	810	4.0	*this work*
Au_9Ag_4	13	~775 [a]	~3 [a]	[15]
$Au_{10}Ag_2$	12	971	4.9	[16]
$Au_{16}Cu_6$	22	720	95.6	[17]

[a] Approximate values estimated from the figures and discussion provided in the reference.

3. *References*

[1] E. Fossum, Z. Yu, L.-S. Tan, *Arkivoc* **2010**, *2009*, 255-265.

[2] M. Iwasaki, M. A. Bakar, Y. Saito, H. Morita, L. Xu, Y. Shichibu, K. Konishi, *Coord. Chem. Res.* **2026**, *3*, 100022.

[3] CrysAlisPro (Version 1.171.142.184a), Rigaku Oxford Diffraction, 2023.

[4] G. M. Sheldrick, *Acta Cryst.* **2015**, *A71*, 3-8.

[5] G. M. Sheldrick, *Acta Cryst.* **2015**, *C71*, 3-8.

[6] O. V. Dolomanov, L. J. Bourhis, R. J. Gildea, J. A. K. Howard, H. Puschmann, *J. Appl. Cryst.* **2009**, *42*, 339-341.

[7] C. Lee, W. Yang, R. G. Parr, *Phys. Rev. B* **1988**, *37*, 785-789.

[8] A. D. Becke, *J. Chem. Phys.* **1993**, *98*, 5648-5652.

[9] F. Weigend, R. Ahlrichs, *Phys. Chem. Chem. Phys.* **2005**, *7*, 3297-3305.

[10] E. Caldeweyher, C. Bannwarth, S. Grimme, *J. Chem. Phys.* **2017**, *147*, 034112.

[11] TURBOMOLE V7.6 2021, A Development of the University of Karlsruhe and Forschungszentrum Karlsruhe GmbH, 1989-2007, TURBOMOLE GmbH, since 2007; available from http://www.turbomole.com.

[12] D. Andrae, U. Häußermann, M. Dolg, H. Stoll, H. Preuß, *Theor. Chim. Acta* **1990**, *77*, 123-141.

[13] R. Ahlrichs, M. Bär, M. Häser, H. Horn, C. Kölmel, *Chem. Phys. Lett.* **1989**, *162*, 165-169.

[14] A. E. Reed, R. B. Weinstock, F. Weinhold, *J. Chem. Phys.* **1985**, *83*, 735-746.

[15] Y. Zhang, W. Zhang, T. S. Zhang, C. Ge, Y. Tao, W. Fei, W. Fan, M. Zhou, M. B. Li, *J. Am. Chem. Soc.* **2024**, *146*, 9631-9639.

[16] Z. Lei, Z. J. Guan, X. L. Pei, S. F. Yuan, X. K. Wan, J. Y. Zhang, Q. M. Wang, *Chem. Eur. J.* **2016**, *22*, 11156-11160.

[17] W.-Q. Shi, L. Zeng, R.-L. He, X.-S. Han, Z.-J. Guan, M. Zhou, Q.-M. Wang, *Science* **2024**, *383*, 326-330.
